# Cocaine Use and Liver Disease are Associated with All-Cause Mortality in the Miami Adult Studies in HIV (MASH) Cohort

**DOI:** 10.21767/2471-853X.100036

**Published:** 2016-11-07

**Authors:** Adriana Campa, Sabrina Sales Martinez, Kenneth E Sherman, Joe Pedro Greer, Yinghui Li, Stephanie Garcia, Tiffanie Stewart, Boubakari Ibrahimou, O. Dale Williams, Marianna K. Baum

**Affiliations:** 1 Florida International University, R Stempel College of Public Health and Social Work, Miami, FL, USA; 2 University of Cincinnati, College of Medicine, Department of Internal Medicine, Cincinnati, Ohio, USA; 3 Herbert Wertheim College of Medicine, Florida International University, Miami, FL, USA

**Keywords:** Cocaine, HIV, Mortality, Liver disease, Oxidative stress

## Abstract

**Objective:**

Liver disease is a frequent cause of morbidity and mortality in HIV infection. We examined the relationship of cocaine use, liver disease progression and mortality in an HIV-infected cohort.

**Methods:**

Consent was obtained from 487 HIV+ participants, a subset of the Miami Adult Studies on HIV (MASH) cohort. Participants were eligible if they were followed for at least two years, completed questionnaires on demographics and illicit drug use and had complete metabolic panels, CD4 cell counts and HIV-viral loads. FIB-4 was calculated and cut-off points were used for staging liver fibrosis. Death certificates were obtained.

**Results:**

Participants were 65% men, 69% Black and 81% were on ART at recruitment. Cocaine was used by 32% of participants and 29% were HIV/HCV co-infected. Mean age was 46.9 ± 7.7 years, mean CD4 cell count was 501.9 ± 346.7 cells/μL and mean viral load was 2.75 ± 1.3 log_10_ copies/mL at baseline. During the follow-up, 27 patients died, with a mortality rate of 28.2/1000 person-year. Cocaine was used by 48% of those who died (specific mortality rate was 13/1000 person-year). Those who died were more likely to use cocaine (HR=3.8, P=0.006) and have more advanced liver fibrosis (HR=1.34, *P*<0.0001), adjusting for age, gender, CD4 cell count and HIV-viral load at baseline and over time. Among the HIV mono-infected participants, cocaine users were 5 times more likely to die (OR=5.09, P=0.006) than participants who did not use cocaine.

**Conclusion:**

Cocaine use and liver fibrosis are strong and independent predictors of mortality in HIV infected and HIV/HCV co-infected adults. Effective interventions to reduce cocaine use among people living with HIV (PHLW) are needed.

## Introduction

As antiretroviral therapy (ART) increases the lifespan of people living with HIV (PLWH), the risk for liver cirrhosis and liver carcinoma increases [1]. Liver disease is one of the predominant causes of non-AIDS related death in HIV-infected patients [2–4] due to the HIV virus itself [5], high prevalence of co-infection with the hepatitis C virus (HCV) [6, 7] and substance abuse [6, 8]. HIV infection is implicated in the development of hepatic fibrosis even in the absence of hepatitis B and C, alcohol use and ART [5]. Survival rates of PLWH, who do not use illicit drugs, are significantly higher compared to those who use drugs even in settings with access to ART [4]. Moreover, HIV infected drug users are at a high risk for HCV co-infection [9] which is associated with increased rates of liver fibrosis, higher rates of cirrhosis, and more rapid progression to end-stage liver disease [10–12].

Despite promising new interventions to address HCV co-infection [13], cocaine use [8, 14] continues to be of interest due to its fibrogenic potential [15]. In addition, HIV infected illicit drug users have lower access to HIV and HCV treatments [16–19] and are at greater risk for treatment failure due to lower adherence to treatment [20]. In HIV/HCV co-infected patients, therapeutic cure of HCV infection may not reduce or reverse hepatic fibrosis and its complications [21, 22]. Patients may not achieve sustained virological response (SVR), may develop occult Hepatitis C [23], which will affect immune recovery [24, 25], may decompensate due to drug and alcohol abuse [26], or develop hepatic malignancy, even after HCV is controlled [27].

Liver injury, regardless of the underlying etiology, is associated with hepatic fibrosis, a wound healing process characterized by excessive deposition of the extracellular matrix and scar formation [28], leading to increased morbidity and mortality [29]. HIV infection is directly implicated in the development of hepatic fibrosis even in the absence of other strong risk factors such as hepatitis B and C infection, alcohol abuse and ART use [5].

Cocaine activates latent HIV-1 infection, increases HIV viral load [8, 30, 31], depresses immune response, [14, 30, 32–35] and accelerates HIV disease progression [8, 30, 32]. Cocaine also activates cytokines associated with progression of liver fibrosis as observed in *in vitro* studies [8, 14]. In this study, we examined the relationship of cocaine use, HIV/HCV co-infection, and progression of liver fibrosis and mortality in an HIV-infected cohort. The objectives were to determine (1) the association of cocaine use and degree of liver fibrosis at baseline and over time in a clinically relevant progression of liver disease; and (2) whether cocaine use and stage of liver fibrosis were associated with mortality during a follow-up. The findings from this investigation suggest that cocaine use facilitates progression of liver fibrosis and mortality in HIV mono-infected and HIV/HCV co-infected adults. Our longitudinal findings are important, because they identify factors and clinical correlates of morbidity and mortality in HIV infected cocaine users, which have the potential to guide the development of effective interventions [16, 36–38].

Since cocaine is the major drug of abuse in our Miami Adult Studies on HIV (MASH) cohort, and drug rehabilitation has largely been unsuccessful in attaining lasting cocaine abstinence [39–42], the results from this study can contribute to a better understanding of the clinical consequences of cocaine use to accelerate the development of targeted programs which might delay mortality, [8, 43, 44] a public health priority [16].

## Methods

### Study design

This was an observational longitudinal study of 487 HIV+ participants followed for at least two years to assess cocaine use, changes in liver fibrosis measured by the liver fibrosis index (FIB-4) and mortality. Participants were a subset of the more than 800 participants from the MASH cohort that has been followed for the last ten years. This study was approved by the Florida International University Institutional Review Board. Appropriate written informed consent was obtained from all participants and clinical research was conducted in accordance with guidelines for human experimentation as specified by the US Department of Health and Human Services and/or authors’ institutions.

To qualify for this investigation, participants needed to be 18–59 years old, have HIV sero-positivity confirmed by medical documentation, and drug use determined by a drug-frequency questionnaire and confirmed by urine toxicology. Participants with uncontrolled diabetes, cirrhosis, hepatitis B infection and kidney failure were excluded. History of HIV infection and hepatitis A, B or C was obtained from the medical record review.

After obtaining informed consent and screening for eligibility, participants’ demographic, medical and drug-related information was obtained from the MASH cohort study charts. Patients were followed for a median of 4.3 years IQR (1–1.08 years). Data for parameters of liver fibrosis were obtained from the participants’ at baseline and 24 month visits. Mortality follow-up was conducted from the study baseline for 6 years, and time of diagnosis was obtained from the medical charts. During the MASH visits, a physical examination was completed and anthropometrics were measured. After overnight fasting, blood samples were obtained for complete blood cell count and blood chemistry that included the liver enzymes alanine aminotransferase (ALT) and aspartate aminotransferase (AST), and platelet counts (PLT) to estimate the fibrosis index (FIB-4). CD4 cell count and HIV viral load were obtained from the participants’ medical records.

## Assesments

### Body mass index

Weight and height were obtained in participants wearing light clothing and no shoes utilizing a standard scale calibrated prior to each measurement. Height was measured with the participant’s heels touching the base of the vertical board of the stadiometer. The moveable headboard was brought to the most superior point on the head with sufficient pressure to compress the hair. Body mass index (BMI) was calculated using the standard formula that divides weight in kilograms by the square of height in meters (kg/m^2^).

### Medical history and review of medical records

Medical history included medical visits, currently prescribed medications, past and current use of ART and adherence in the previous 6 months; a review of records was used to verify prescriptions, determine changes in ART, and confirm diagnosis.

### Staging liver disease

A non-invasive measure to estimate liver fibrosis, the fibrosis index (FIB-4), was calculated using routine laboratory tests as follows: [age (years) × AST (U/L)]/[PLT (10^9^ cells/L) × ALT^1/2^ (U/L)] [45]. Liver fibrosis stages F0–F1 correspond to FIB-4<1.45; F2–F3 to ≥ 1.45 but ≤ 3.25; and F4 to FIB-4 >3.25 [46]. At a cut-off point of <1.45 (equivalent to Metavir stage F0–F1), the negative predictive value to exclude advanced fibrosis (Metavir stage F4) was 90% with a sensitivity of 70%. A cut-off of >3.25 (equivalent to Metavir stage F-4) had a positive predictive value of 65% and a specificity of 97% to predict advanced disease [46].

### Substance abuse questionnaires

Drug, alcohol and tobacco use questionnaires were administered by trained and certified interviewers. The drug use questionnaires were validated against blood metabolites and urine screening [30] and described previously [31, 47]. Cocaine users were defined as participants who admitted to cocaine use by self-report and/or had at least one positive urine toxicology (N=156). Cocaine use frequency was captured through validated questionnaires and scored as: 0=never, 1=once a month, 2=once a week, 3=3–4 times a week, 4=daily and 5=more than daily. Frequency of use was obtained from participants who admitted using during their assessment visits (N=80). Alcohol use was obtained using the validated and standardized Alcohol Use Disorders Identification Test (AUDIT) questionnaire that detects frequency of use, hazardous and dependent drinking, and binge drinking [48].

### Urine toxicology

Urine was collected under observation at each study visit. The American Biomedica Rapid Drugs kits were used to confirm self-report for cannabinoids, cocaine, methadone, methamphetamines and opiates.

### Death certificates

Death certificates were obtained from the State of Florida Department of Vital Statistics through a research agreement to verify time and cause of death and to determine whether the death was HIV-related.

## Statistical Analysis

To characterize the population, we used descriptive statistics that were expressed as mean ± standard deviation (SD) or as percent of population. Significance was defined as P<0.05. The Cox proportional hazard model was employed to derive adjusted hazard ratios after testing for non-violation of the proportionality assumption in each case. We confirmed this by plotting the log-negative-log of the Kaplan-Meier estimates of the survival function versus the log of time using ‘R’ statistical software, version 3.1.1. The resulting curves were parallel [49]. The computed hazard was determined by:

h (t)=h0 (t) × exp {b1 × 1+b2 × 2+...+bpxp} where the hazard function is represented by h (t) and is determined by a group of covariates (x1, x2 ..., xp) whose effect is measured by the size of the individual coefficients (b1, b2, ..., bp) and t is the survival time. All statistical analysis were performed using SAS statistical software, version 9.4. Mixed models were used to assess the effect of cocaine on liver fibrosis over two years. Participants in both groups were in a waiting list for ART at baseline, depending on when they were diagnosed, but were referred for treatment during participation. The great majority received treatment within one year of starting in the study, as ART became more accessible in Miami-Dade. The analyses were adjusted for viral load at baseline and over time, because viral load control was a stronger predictor of disease progression and mortality than just receiving ART. All the analyses were also adjusted for age, gender, CD4 cell count and HIV-viral load at baseline and over time.

## Results

### Characteristics of the population

There were a total of 487 participants, a subset of the MASH cohort, who were followed for the markers of liver disease progression and death. Participants were mostly men (65%), African Americans (68%) and receiving ART (81%) at baseline. Thirty-two percent used cocaine and 29% were HIV/HCV co-infected. Mean age was 46.9 ± 7.7 years, mean CD4 cell count was 501.9 ± 346.7 cells/μL and mean viral load was 2.75 ± 1.3 log_10_ copies/mL at baseline (Table 1). During the follow-up, 27 patients died, with a mortality rate of 28.2/1000 person-year. Thirteen of these participants (48%) reported cocaine use during the study with a specific mortality rate of 13/1000 person-year. Participants who used cocaine had significantly lower Body Mass Index (BMI), although the mean BMI was still in the “overweight” range (26.9 ± 5.4 kg/m^2^ cocaine users vs. 27.67 ± 5.1 kg/m^2^ among non-users, P=0.026). Fewer cocaine users received ART at baseline (75% vs. 84%, P<0.001) and they had higher HIV viral load than the participants who did not use cocaine (2.97 ± 1.4 log_10_ copies/mL vs. 2.64 ± 1.3 log_10_ copies/mL, P=0.001). Significantly fewer Hispanics but more African Americans used cocaine (Table 1).

### Effect of cocaine use on liver fibrosis in HIV/HCV co-infected and HIV mono-infected PLWH

Table 2 shows the comparison between cocaine users (32%) and non-users among HIV/HCV co-infected and HIV mono-infected participants on advancing liver disease progression over a period of two years. Both HIV/HCV co-infected and HIV mono-infected cocaine users had significantly faster liver fibrosis progression than non-users. Cocaine use among those who were HIV/HCV co-infected was associated with 0.44 unit increase in FIB-4 (β=0.44, SE=0.22, P=0.049) and among the HIV mono-infected with an increase of 0.23 units in FIB-4 (β=0.23, SE=0.11, P=0.033), as compared to cocaine non-use over a two-year period. Moreover, cocaine users, both HIV mono-infected and HIV/HCV co-infected had more than twice the risk of advancing liver fibrosis from FIB-4 <1.45 to >3.25, equivalent to clinically significant 2-Metavir stages (OR=2.52, 95% CI 1.15, 5.54, P=0.021), compared to cocaine nonusers (not shown). In addition, a higher proportion of HIV/HCV co-infected cocaine users progressed the equivalent of 2-Metavir stages in two years, compared to HIV/HCV co-infected cocaine non-users (62.5% vs. 30.4%, P<0.0001).

HIV/HCV co-infected and HIV mono-infected participants who used cocaine daily or more than daily, had higher FIB-4 compared to moderate cocaine users and non-users over time (β=0.6, SE=0.3, P=0.03). In addition, daily or more than daily use of cocaine was a significant predictor of FIB-4 >1.45 in the range of moderate or more severe fibrosis over time (β=0.2, SE 0.1, P=0.02) among HIV mono-infected participants.

### Effect of cocaine and liver fibrosis on mortality

During the 6-year follow-up, 27 patients died, 13 of whom (48%) reported cocaine use during the study. The mortality was higher among cocaine users (13/156, 8.33%) compared to non-users (14/331, 4.22%, P=0.003). Twelve participants died among those who were HIV/HCV co-infected (12/140, 8.6%) and 15 participants among those who were HIV mono-infected (15/347, 4.3%). The specific rate of mortality for participants who were HIV/HCV co-infected was 12.32/1000 patient-year. Table 3 shows that HIV/HCV co-infection significantly predicted death (P=0.035) and those who were co-infected were 2.4 times more likely to die during the study. HIV mono-infected participants who used cocaine were 5 times more likely to die (HR=5.1, CI 95%: 1.6, 16.1, P=0.006) controlling for age, gender and HIV viral load at baseline (Table 3). In Table 4, in a Multiple Cox Regression Model, those who died were more likely to use cocaine (HR=3.8, CI 95%: 1.5, 9.8, P=0.006), have higher FIB-4 at baseline (HR=1.34, 95% CI: 1.18, 1.52, P<0.0001), and lower CD4 cell count (HR=0.94, 95% CI: 0.88, 0.99, P=0.0194) than those who survived, adjusting for age, gender and HIV viral load at baseline and over time. The majority of death certificates (19/27, 70.4%) had multiple causes of death, with 17 certificates listing HIV/AIDS as a major cause of death, followed with 10 with diagnosis of cardio-respiratory arrest (Table 5).

**Figure 1:**
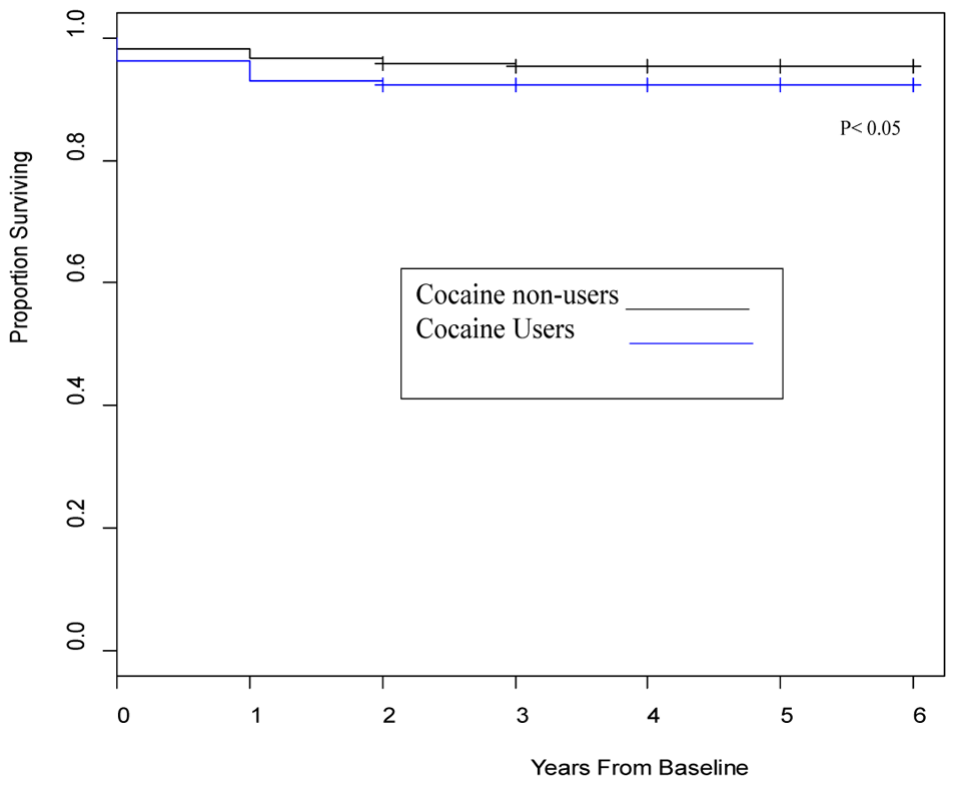
Kaplan-Meier curves comparing the survival of cocaine user assessed by self-report and/or urine toxicology or both with that of non-users from the study baseline visit. **Interpretation:** Cocaine users had significantly lower survival time than participants who did not use cocaine when followed from study baseline for 6 years, P<0.05.

**Figure 2:**
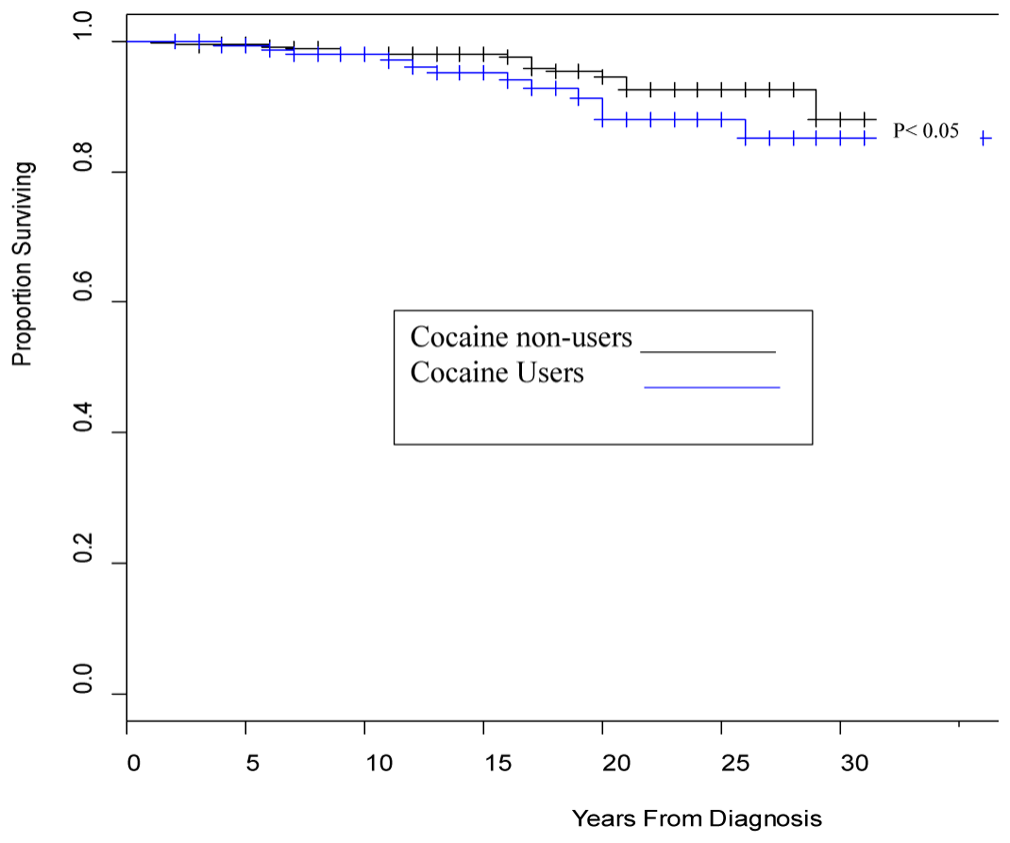
Kaplan-Meier survival curves comparing the survival of cocaine use assessed by either self-report, urine toxicology or both with that of non-users from the reported years since diagnosis. **Interpretation:** The Kaplan-Meier curve graphs the survival function over the number of years since diagnosis. The survival function describes the probability of surviving past year *y*. For example, the probability of surviving past 20 years after being diagnosed is about 94.5% for those who did not use cocaine and about 88% for those who use cocaine assessed by either self-report or urine toxicology. A drop in the graph (vertical line) represents death while the vertical ticks through the horizontal lines represent censored subjects, or subjects who did not die within the time frame of the study.

**Figure 3:**
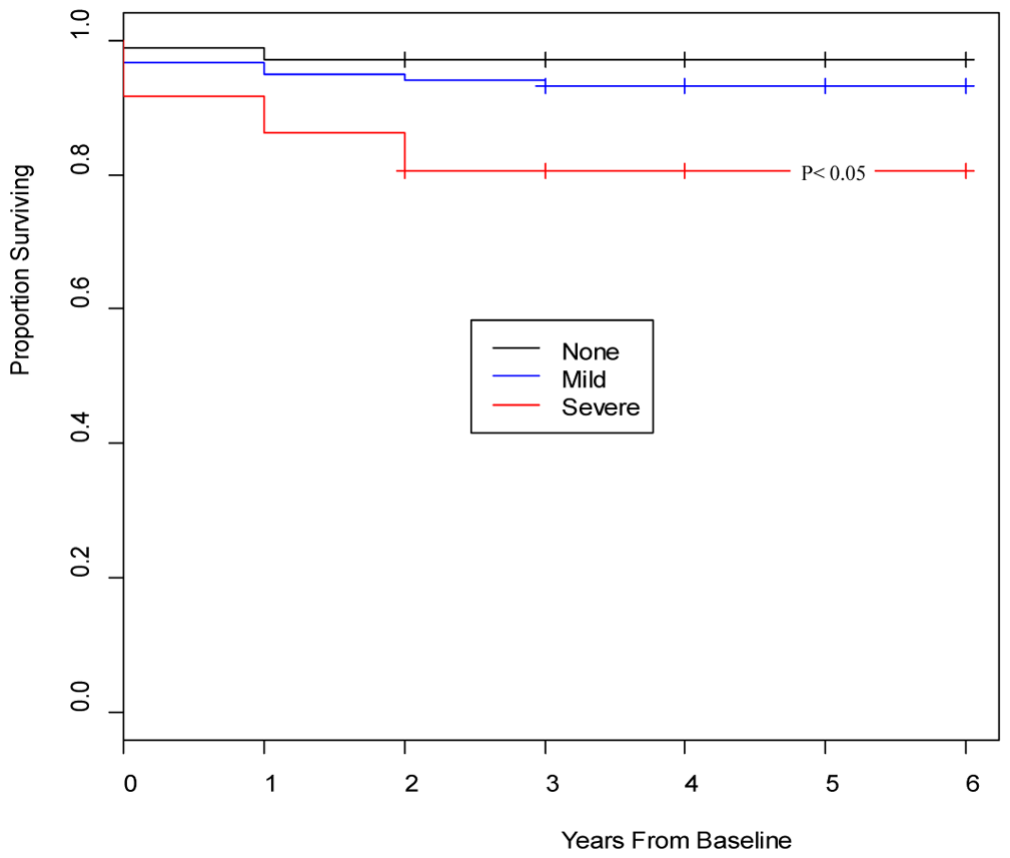
Kaplan-Meier survival curves comparing the survival of participants by severity of liver fibrosis estimated by FIB-4 at baseline to the end of study. **Interpretation:** From baseline to the end of the study, the probability of surviving is about 97% for those with no liver fibrosis and approximately 93% for those with mild to moderated liver fibrosis and about 80% for those with severe liver fibrosis.

**Figure 4:**
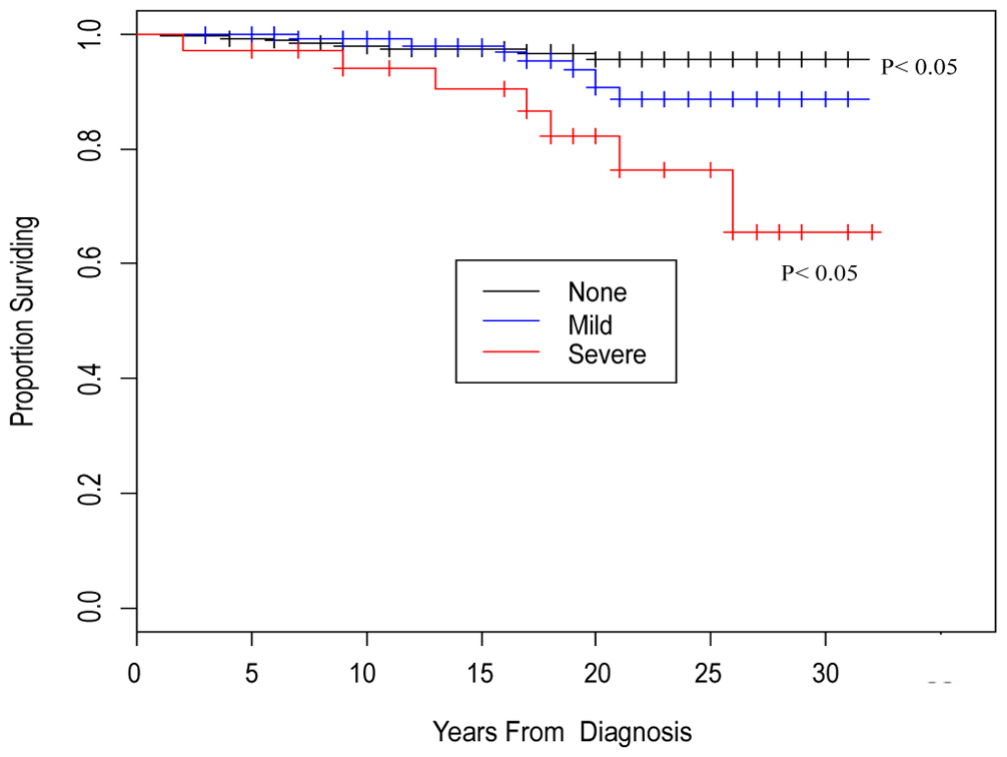
Kaplan-Meier curve comparing survival by severity of liver fibrosis estimated by FIB-4 at baseline from the reported years since HIV diagnosis **Interpretation:** The Kaplan-Meier curve graphs the survival function over the number of years since diagnosis. The survival function describes the probability of surviving past year *y*. For example, the probability of surviving past 20 years after being diagnosed is about 96% for those with no liver disease (F0–F1), about 91% for those with mild liver disease (F2–F3) and roughly 76% for those with severe liver disease (F4–F5) A drop in the graph (vertical line) represents death while the vertical ticks through the horizontal lines represent censored subjects, or subjects who did not die within the time frame of the study.

Figures 1–4 depict the Kaplan-Meier Curves comparing the survival of cocaine users compared with non-users and survival by stage of liver fibrosis. The outcome event is time of death confirmed by a death certificate. Two time frames are considered: from the study baseline to the event during a 6 year follow-up in Figure 1 and from the time of HIV diagnosis obtained from medical charts to death in Figure 2. The Kaplan-Meier curves in both figures indicate a significantly shorter survival time for participants who used cocaine than those who did not use cocaine (P<0.05). Figures 3 and 4 compare survival by different stages of liver fibrosis estimated by FIB-4 for the two years and show that participants who had more severe liver disease had significantly higher rates of survival than those who had less severe liver disease (P<0.05).

## Discussion

Liver disease is a predominant cause of morbidity and mortality in HIV infection [2–4]. This study has shown the association between cocaine use and increased likelihood of liver disease progression and death in people living with HIV and those co-infected with HIV and HCV. Although the mortality rate of this group is relatively high (28.24 per 1000 person-year), it is comparable to that of other HIV infected cohorts that include drug users [50, 51]. Cocaine users, both HIV/HCV co-infected and HIV mono-infected, had significantly faster liver fibrosis progression and higher rates of mortality than participants who did not use cocaine. The association between cocaine use and moderate to severe liver fibrosis progression among the HIV mono-infected participants was even stronger than in the combined cohort of HIV/HCV co-infected and HIV mono-infected participants, and the association of cocaine use with mortality was higher (Table 3). Among these MASH cohort participants, the mortality rate was higher in the cocaine users compared to cocaine non-users (8.33% vs. 4.22%, *P*=0.003, (Table 1), which is in agreement with other studies showing increased rate of death among cocaine users. [52].

In previous reports [30, 31], we established the association of cocaine use with HIV disease progression [30] and suggested mechanisms [31] for this effect. The findings of the present study support this association and show that both cocaine use and CD4 cell counts, a marker of disease progression, remain strongly significant as independent predictors of death in the multivariate model (Table 3). In this study, cocaine users had significantly higher HIV viral load at baseline (2.97 ± 1.4 vs. 2.64 ± 1.3, P<0.001 (Table 1), which has been previously shown to affect liver enzyme elevations and more advanced hepatic fibrosis. Thus, our observations support earlier findings of other investigators indicating an association between increased HIV viral load and liver fibrosis [5], most likely due to an infection of hepatocytes by HIV, inducing liver fibrogenesis [53]. In addition, Dillon et al. [54] reported that cocaine activated viral replication in human monocyte-derived macrophages and intra-hepatic infiltration of HIV-specific CD8 and has been suggested as a mechanism for rapid liver fibrosis in HIV/HCV co-infected patients [55], particularly in those who use cocaine. We have shown in this current study that HIV-infected cocaine users not only demonstrate higher HIV viral load, but also fewer percent of these participants report taking ART. ART has been documented to slow liver disease progression, by both biopsies and clinical events, including lowering inflammation, lowering risk of hepatic decompensation and death [56]. The results of this study support the earlier findings by other investigators showing that cocaine use is independently associated with failure to achieve virologic suppression in HIV infected patients [57]. On the other hand, our finding that cocaine use was associated with lower BMI in this study, may be protective of hepatic fibrogenesis, as liver disease has been previously shown to be associated with obesity [58].

The faster liver disease progression and elevated mortality observed among cocaine users compared with non-users in this study may be due to cocaine users having higher viral load and lower use of ART, but also to direct damage to the liver by cocaine, which may range from just elevated hepatic enzymes to hepatic necrosis precipitated by high cocaine doses [59]. In animal models, cocaine has been shown to act as a hepatotoxin, with the cytochrome P-450 dependent N-oxidative pathway exerting direct cytotoxic effects through oxidative damage causing liver injury [60]. The net amount of reactive species seems to determine the severity of hepatic lesions [60]; thus, these findings also may explain the associations of higher frequency of cocaine use with faster liver disease progression as the frequency of cocaine use increased.

Another unique finding was that faster liver disease progression and higher rate of cocaine-related mortality occurred in both HIV/HCV co-infected and HIV mono-infected participants. While the effect of high frequency of cocaine use was significantly associated with the progression of liver disease and mortality in HIV mono-infected participants, HCV co-infection was most likely a stronger driver of liver fibrosis among the HIV/HCV co-infected participants [61].

In this study we used a non-invasive measure of liver fibrosis, the FIB-4 index, which has been suggested as an alternative to liver biopsy in PLWH, due to significant limitations such as sampling errors, observer variation and the risk for severe complications such as severe bleeding [62, 63]. The FIB-4 index has been validated in HIV/HCV co-infected adults [45, 64]. Recently, FIB-4 was found to be a better predictor of overall death and liver-related events than liver biopsy in HIV/HCV co-infected individuals [64]. Liver biopsy is not routinely performed in HIV mono-infected individuals without any liver related disease, therefore non-invasive measures of liver damage are appropriate for this population and have been performed in other studies with HIV mono-infection.

## Limitations

This study determined the associations between cocaine use, the progression of liver disease and fibrosis, and mortality among HIV/HCV co-infected and HIV mono-infected PLWH. While the participants had a long-term follow-up, the study was observational and therefore it is not possible to draw causative inferences. Our findings are also limited by the specific characteristics of the population: people living with HIV and substance abuse in Miami, which may be very different than other populations of PLWH nationally and internationally.

## Conclusion

Cocaine use increased the likelihood of progression of liver fibrosis and death in HIV mono-infected and HIV/HCV co-infected participants. Findings from this study suggest that cocaine use in PLWH has deleterious effects on viral control, progression of liver disease and survival and that the detrimental impact of cocaine use remains even when co-infection with HCV is not present. These results suggest the need to investigate potential mechanisms of action of HIV and cocaine on liver fibrosis and survival in human studies and for developing effective intervention programs in people living with HIV to increase access and adherence to treatment, reduce progression of liver fibrosis and prevent early death.

## Figures and Tables

**Table 1:** Characteristics of the population.

Characteristics	Total (N=487)	Cocaine Users (N=156) 2.0%	Cocaine non-users (N=331)	*P*-value

**Gender (%)**				
Male	65%	69%	63%	
Female	35%	31%	37%	0.101

**Age (years)**	46.9 ± 7.7	46.4 ± 7.5	47.2 ± 7.8	0.189

**Ethnicity (%)**				
White	6.8%	6.7%	6.9%	0.939
Hispanic	18%	10.1%	21.4%	<0.001*
African American	67.8%	73.2%	65.8%	0.023*
Other	7.2%	10.0%	5.9%	0.082

**Receiving ART at baseline**	81%	75%	84%	0.001*

**HIV/HCV co-infected**	28.8%	28%	29%	0.619

**BMI kg/m^2^**	27.4 ± 5.2	26.9 ± 5.4	27.67 ± 5.1	0.026*

**CD4 cells/μL**	501.9 ± 346.7	508.8 ± 377.8	499.4 ± 332.3	0.697

**HIV viral load log_10_ copies/mL**	2.75 ± 1.3	2.97 ± 1.4	2.64 ± 1.3	<0.001*

**FIB-4 ≥ 1.45**	63.9%	64.2%	65.4%	0.796

**Deaths**	5.54% (27/487)	8.33% (13/156)	4.22% (14/337)	0.003*

* Statistically Significant, P<0.05

**Table 2:** Mixed model estimates of the effect of cocaine on liver fibrosis over a period of two years.

Four Study Groups		β	SE*	P-value
HIV/HCV using cocaine (N=58)	HIV/HCV no cocaine use (N=60)	0.44	0.22	0.049**
HIV mono-infected using cocaine use (N=94)	HIV mono-infected no cocaine use (N=76)	0.23	0.11	0.033**

Controlled for age, sex and baseline CD4 cell count and viral load

* SE=Standard Error

** Statistically Significant, P<0.05

**Table 3:** Logistic regression of predictors of death in PLWH*.

Predictors	Odds Ratio	95% CI**	P-value
**(N=487) HIV Mono-infected and HIV/HCV Co-infected Persons**			
Cocaine Use	2.8	1.14, 6.8	0.024†
HIV/HCV co-infection	2.4	1.06, 5.4	0.035†
FIB4 ≥ 1.45 at baseline	3.7	1.23, 11.2	0.019†
**(N=347) HIV-Mono-infected Persons Only**			
Cocaine Use	5.1	1.6, 16.1	0.006†
FIB ≥ 1.45	1.53	1.2, 1.9	<0.001

* All models were controlled for age, gender and HIV viral load at baseline

** 95% Confidence Intervals

† P-value<0.05

**Table 4:** Multiple cox regression models for cocaine as predictors of mortality.

Variable	Parameter Estimate	Standard Error	Hazard Ratio	95% CI	P-value
Cocaine use (assessed by self-report & urine toxicology)	1.33	0.481	3.80	1.5, 9.8	0.006
CD4 cell count (cells/μL)	−0.07	0.029	0.94	0.88, 0.99	0.0194
FIB-4 index score	0.30	0.063	1.34	1.18, 1.52	<0.0001

* P-value<0.05

† All univariate models were controlled for age, gender and HIV viral load at baseline and over time
The hazard of dying is 3.80 times higher for individuals who used cocaine (n=156) as compared to those who did not use cocaine (HR=3.80, P=0.0056) For every one unit increase in the FIB-4 index score there is a 34.3% increase in the adjusted mortality hazard (HR=1.343, P<0.0001) For every one unit increase in CD4 cell count there is a 6.5% decrease in the adjusted mortality hazard (HR=0.935, P=0.0100)

**Table 5:** Frequency of causes of death according to death certificates.

Cause of Death	Frequency*
HIV/AIDS	17
Cardiovascular Disease	13
Respiratory Failure	5
Acute Liver Failure	3
Acute Encephalopathy	2
Acute Renal Failure	2
Pneumonia	2
Septic Shock, Sepsis	2
TB	1
Cocaine and Ethanol Toxicity	1
End Stage Lung Cancer	1
Acetaminophen Overdose	1
Gunshot Wound to the Neck	1
Wasting Syndrome	1
Cerebral Amyloid Angiopathy	1
Total	53

* 70.4% (19/27) contained more than one cause of death, 63% (17/27) listed HIV/AIDS one of the causes of death
